# Ultrahigh-temperature tensile creep of TiC-reinforced Mo-Si-B-based alloy

**DOI:** 10.1038/s41598-018-28379-w

**Published:** 2018-07-11

**Authors:** Shiho Yamamoto Kamata, Daiki Kanekon, Yuanyuan Lu, Nobuaki Sekido, Kouichi Maruyama, Gunther Eggeler, Kyosuke Yoshimi

**Affiliations:** 1 Department of Materials and Science, Graduate School of Engineering, Tohoku University, Sendai, 980-8579 Japan; 2 Institute for Materials, Ruhr-University Bochum, Universitaetsstrasse 150, D-44780 Bochum, Germany; 3 Present Address: Ferroalloys Sect. 1, Ferroalloys, Non-Ferrous & Precious Metals Dept., Metals & Coal Division, Sojitz Corporation, Tokyo, 100-8691 Japan

## Abstract

In this study, the ultrahigh-temperature tensile creep behaviour of a TiC-reinforced Mo-Si-B-based alloy was investigated in the temperature range of 1400–1600 °C at constant true stress. The tests were performed in a stress range of 100–300 MPa for 400 h under vacuum, and creep rupture data were rationalized with Larson-Miller and Monkman-Grant plots. Interestingly, the MoSiBTiC alloy displayed excellent creep strength with relatively reasonable creep parameters in the ultrahigh-temperature range: a rupture time of ~400 h at 1400 °C under 137 MPa with a stress exponent (*n*) of 3 and an apparent activation energy of creep (*Q*_*app*_) of 550 kJ/mol. The increasing rupture strains with decreasing stresses (up to 70%) and moderate strain-rate oscillations in the creep curves suggest that two mechanisms contribute to the creep: phase boundary sliding between the hard T_2_ and (Ti,Mo)C phases and the Mo_ss_ phase, and dynamic recovery and recrystallization in Mo_ss_, observed with orientation imaging scanning electron microscopy. The results presented here represent the first full analysis of creep for the MoSiBTiC alloy in an ultrahigh-temperature range. They indicate that the high-temperature mechanical properties of this material under vacuum are promising.

## Introduction

Many key technologies rely on systems that operate at elevated temperatures, ranging from energy conversion systems in automotive applications and power plants to propulsion systems in aircraft engines and rockets. In all of these systems, thermal energy is transformed into mechanical energy. Some critical high-temperature components limit the service life of such systems; these rely on high-temperature materials that can resist mechanical loads at high temperatures. High-temperature processes and critical high-temperature components can differ in many ways; however, their common aspect is a steady driving force for increased thermal efficiencies, as expressed by the second law of thermodynamics^1,2^. Therefore, researchers in this field aim to develop materials that can operate at higher service temperatures.

At present, Ni-based single crystal superalloys, which are used for turbine blades, are the most commercially successful class of metallic high-temperature materials^3,4^. However, they cannot be used at temperatures above 1200 °C. The upper temperature limit for use of a material depends on its melting point, which is 1455 °C for Ni^5^. For Ni-based superalloys, the temperature at which the strengthening γ’-phase dissolves (γ’-solvus^3^), i.e. approximately 1200 °C, also needs to be considered. Because single-phase alloys do not exhibit good high-temperature strength, they need to be strengthened with fine particle dispersoids. Refractory metals such as Mo have much higher melting points than Ni (Mo: 2623 °C^5^); therefore, Mo-based alloys are considered attractive candidates for ultrahigh-temperature applications. The Mo-Si-B ternary system has received much attention in the scientific literature^6–10^. However, this system suffers from high-temperature oxidation and needs to be strengthened with more stable particles^11–18^. Research in this field is ongoing. In the past five years, a particle-strengthened system of the MoSiBTiC type^12,18^ has been developed with a composition 65Mo-5Si-10B-10Ti-10C (at%). In this study, this material is referred to as ultrahigh-temperature Mo alloy (MoSiBTiC).

Interest in the high melting point of Mo arises from the fact that vacancy densities scale with the relative distance to the melting point and thus different homologous temperatures, *T*_h_, which expresses the temperature of a material as a fraction of its melting point (*T*/*T*_m_; temperatures in K). At a given temperature, a metal with a lower melting point has a higher *T*_h_ than a metal with a higher melting point; therefore, the former will contain a higher density of thermal vacancies and creep faster. The creep of metallic materials depends on dislocation climb, which in turn depends on the presence of vacancies^19–24^. Creep research in recent decades has shown that there is a need to strengthen metallic alloys with fine stable particle dispersoids^19–24^, which has led to the development efforts discussed above^12,18^.

Creep is a fascinating research subject in itself. It represents a time-dependent plastic deformation of materials and exhibits strong stress and temperature dependence. In general, the creep life is spent at the steady-state creep rate, ${\dot{\varepsilon }}_{{\rm{st}}}$. As a result, the stress and temperature dependence of the creep is often described by an expression that only addresses the steady-state creep rate. This is represented by a combination of a power law and an Arrhenius-type equation as follows:

1 $${\dot{\varepsilon }}_{{\rm{st}}}={\rm{C}}\cdot {\sigma }^{n}\cdot \exp (-\frac{{Q}_{{app}}}{{RT}})$$

where *C* is a constant, *σ* is the uniaxial tensile stress, *n* is the stress exponent, *Q*_*app*_ is the apparent activation energy, and *R* and *T* have their usual meanings. This is applicable for alloys in the ultrahigh-temperature range, particularly the MoSiBTiC alloy considered in this study. In order to determine the creep behaviour of MoSiBTiC, creep tests need to be performed under a protective atmosphere or in a vacuum to prevent oxidation. At present, only a limited amount of constant strain rate and compression creep data exist for temperatures up to 1300 °C. To the best of our knowledge, there is only one publication that has reported tensile creep data at temperatures up to 1300 °C^25^. That study reports stress exponents, *n*, of between 4.3 and 7.1, and an apparent activation energy of creep, *Q*_*app*_, of 360 kJ/mol. The scientific objective of the present study is to provide the first creep data set for a MoSiBTiC alloy. All creep experiments were performed at computer-controlled constant true stress in the temperature range of 1400–1600 °C. The test procedure is documented, and the shapes of individual creep curves as well as the stress and temperature dependence of the minimum creep rate, which is often used instead of the steady-state creep rate, are reported. An effort is made to rationalize the mechanical data based on microstructural observations and creep mechanisms that have been proposed in the literature. This is the first report presenting a tensile creep data set for a Mo-Si-B-based alloy in the ultrahigh-temperature region.

## Results

**Figure 1:**
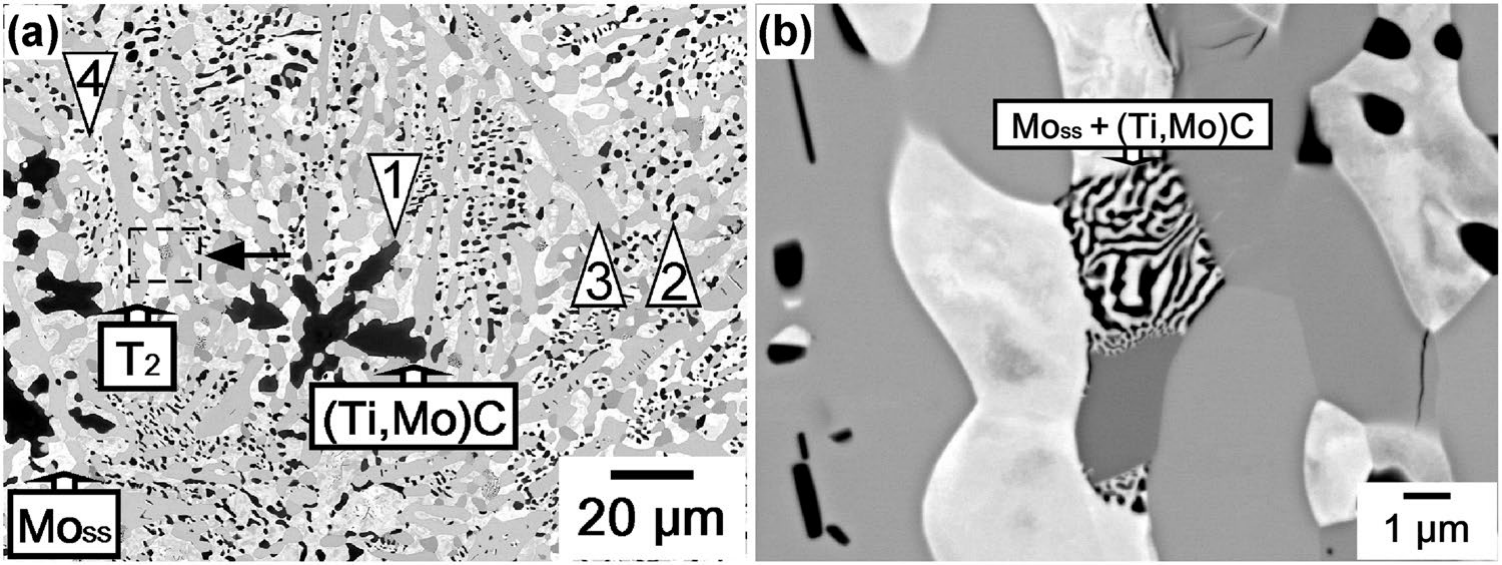
SEM micrographs of the MoSiBTiC alloy prior to creep testing. (**a**) Overview micrograph showing the distribution of phases. (**b**) Higher magnification micrograph showing local microstructural features.

Micrographs of the complex microstructure of the MoSiBTiC alloy used in this study are shown in Fig. 1. The MoSiBTiC alloy mainly consists of three phases: a Mo-rich solid solution Mo_ss_ (Mo base with 2.5 at% Ti and 1.9 at% Si, crystal structure: bcc), which appears as a white contrast; a Mo_5_SiB_2_-type intermetallic compound known as T_2_ (crystal structure: D8_*l*_), which appears grey; and a (Ti,Mo)C-type carbide (crystal structure: NaCl-type), which appears as a black contrast. The large particle marked “1” reveals the dendritic nature of the particle resulting from its formation history during solidification. From Fig. 1, it is evident that the alloy microstructure is heterogeneous on the micron scale. In the overview micrograph shown in Fig. 1(a), regions with coarse (Ti,Mo)C particles (e.g. location 1) exist adjacent to regions with very fine particles (e.g. location 2). The same observation is made for the T_2_ phase, which can be seen to occupy large elongated regions (e.g. location 3) and to form fine dispersoids (e.g. location 4), probably as a result of an eutectic reaction during solidification^12,18^. Compared to the other two phases, the Mo_ss_ phase appears to be more regularly distributed throughout the microstructure. Figure 1(b) is a high-magnification image of the region indicated by the dashed rectangle in Fig. 1(a). The arrow at the centre of the image indicates a fine microstructure, which is probably a result of an eutectoid reaction during cooling from the homogenization temperature. As reported in our previous work^12,13^, a small amount of (Mo,Ti)_2_C was included in an as-cast state of the alloy. Most of (Mo,Ti)_2_C phase, however, decomposes into Mo_ss_ and (Ti,Mo)C phases through an eutectoid reaction during homogenization heat treatment^15,26^; thus, its volume fraction decreases to less than 1%^18^. The volume fractions of the three phases obtained after processing (melting, casting, and homogenization heat treatment) are 46, 36, and 18% for Mo_ss_, T_2_, and (Ti,Mo)C, respectively^18^. From Fig. 1, it is evident that these values can have strong local variations on the micron scale.

**Figure 2:**
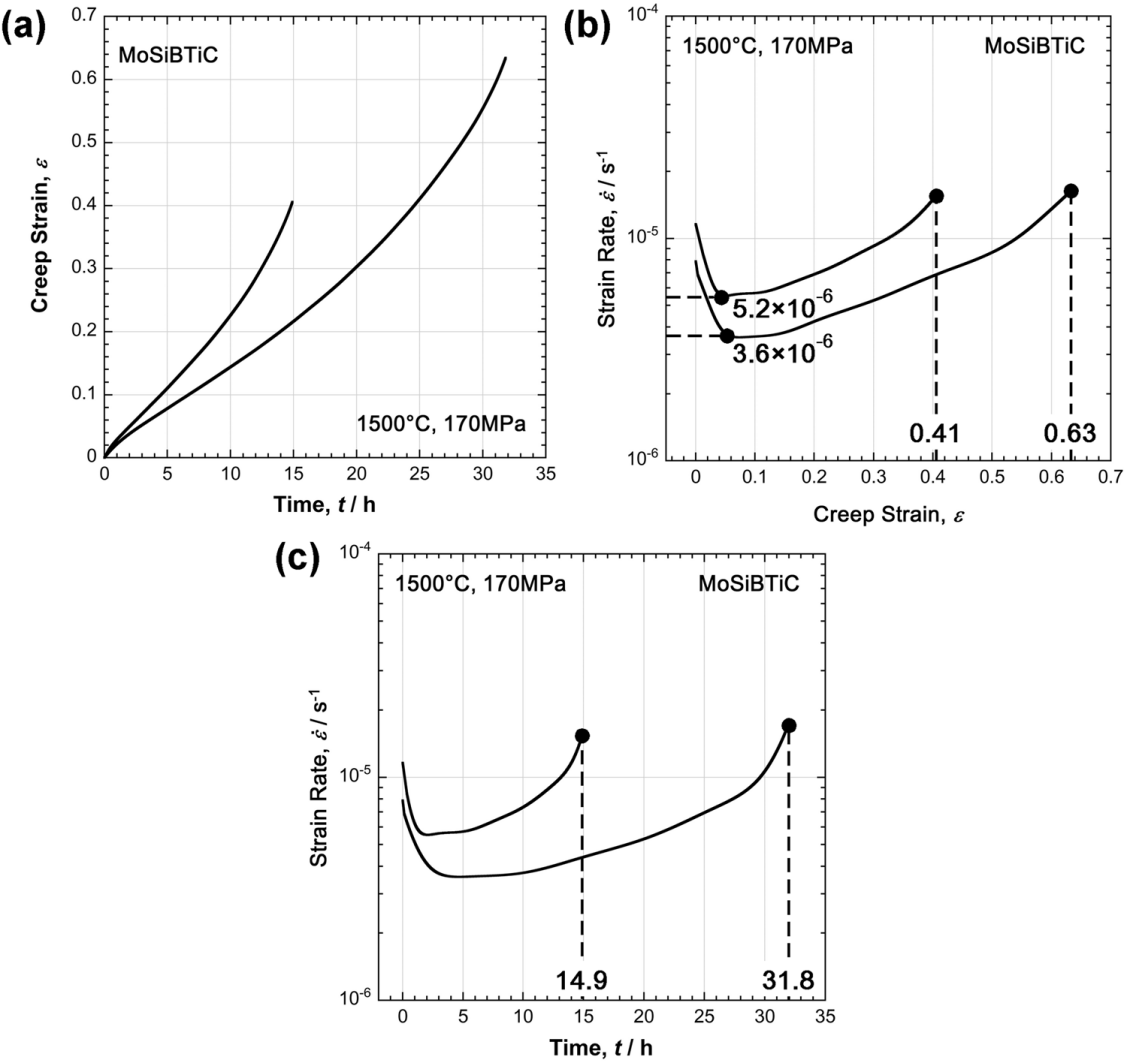
Creep curves recorded for two creep tests at 1500 °C and 170 MPa. (**a**) Creep strain, *ε*, as a function of time, *t*. (**b**) Logarithmic strain rate, $\mathrm{log}\,\dot{\varepsilon }$, as a function of strain, *ε*. (**c**) Logarithmic strain rate, $\mathrm{log}\,\dot{\varepsilon }$, as a function of time, *t*.

Figure 2 shows the creep curves obtained from two creep tests performed at 1500 °C and 170 MPa in this study. In Fig. 2(a), the curves are plotted in the traditional way (*ε*(*t*) curves) as the strain, *ε*, vs. *t* (in h), whereas in Fig. 2(b), the two curves are plotted as the logarithmic creep rate $\mathrm{log}\,\dot{\varepsilon }$, vs. *ε* ($\mathrm{log}\,\dot{\varepsilon }(\varepsilon )$ curves). In Fig. 2(b), the two horizontal dashed lines indicate the logarithm of the minimum creep rates $\dot{\varepsilon }$_*min*_, which only differ by a factor of 1.03. This indicates that the creep test procedure is reproducible. Furthermore, the two rupture strains differ by a factor of 1.5; the higher scatter in the rupture strains is due to the stochastic nature of creep damage accumulation. This is also observed for other engineering materials, such as superalloy single crystals^27^. In Fig. 2(c), the logarithmic creep rates are plotted as a function of time ($\mathrm{log}\,\dot{\varepsilon }(t)$ curves). This plot illustrates the importance of minimum creep rates for engineering, as that is where the material spends most of its creep life.

For a basic analysis of creep, the plot $\mathrm{log}\,\dot{\varepsilon }$ vs. *ε* provides insight into the response of a material to constant stress loading while directly indicating the state of deformation. Table 1 summarizes all the creep tests performed in this study; test temperatures (°C) and stresses (MPa) are listed along with corresponding rupture times (h) and strains (%).

**Table 1:** Overview of creep tests performed until rupture in the present work.

*T*(°C)	*t*_R_ (h)/*ε*_R_ (%); *indicates interrupted experiment: *t* (h)/*ε* (%)						
	100	120	137	170	200	250	300
1400	—	—	397/61	149/51	91.0//42	38.0/31	17.5//20
1500	114/89	67.1/71	44/72 4.3*/2.9*	31.8/63 14.9/41	17.2/68	5.2//52	—
1600	17.6/60	—	5.0/62	4.6/58	1.8/54	0.5/36	—

**Figure 3:**
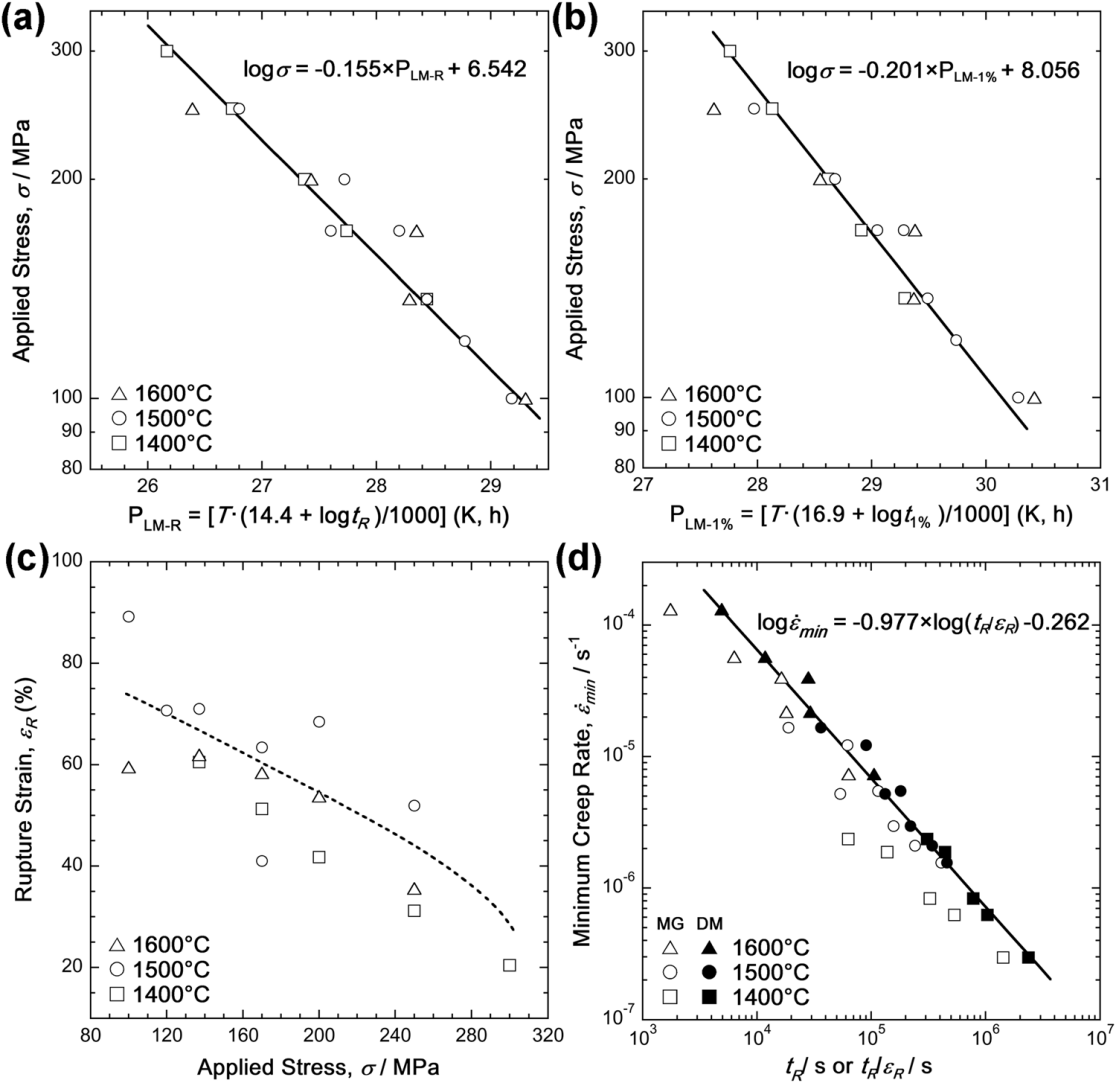
Creep rupture plots for the creep rupture and minimum creep-rate data obtained in this study. (**a**) Larson-Miller plot of rupture data with an optimized constant of 14.4 (stress in MPa, temperature in K, time in h). (**b**) Larson-Miller plot of 1% data with an optimized constant of 16.9 (stress in MPa, temperature in K, time in h). (**c**) General trends showing that rupture strains increase with decreasing stress. (**d**) Monkman-Grant (minimum creep rate vs. rupture time) and Dobeš-Milička plots.

Figure 3 shows the creep test results, which are relevant for engineering. Figure 3(a) displays the creep rupture data from Table 1 in a Larson-Miller plot^28^, in which the applied stress, *σ*, is plotted as a function of the Larson-Miller parameter ${P}_{{\rm{L}}{\rm{M}}{\textstyle \text{-}}{\rm{R}}}=T\cdot ({\rm{a}}+\,{\rm{l}}{\rm{o}}{\rm{g}}\,{{t}}_{{\rm{R}}})$, where a is a fitting parameter. The *P*_LM-R_ parameter represents a temperature-compensated rupture time. This plot brings the three distinct sets of creep rupture data measured at 1400, 1500, and 1600 °C reasonably close to one common master curve. This master curve can be represented by a linear equation of the type:

2 $$\mathrm{log}\,\sigma ={\rm{b}}\cdot {P}_{\mathrm{LM}-R}+{\rm{c}}$$

where *σ* and *P*_LM-R_ are the stress and Larson-Miller parameter, respectively, and b and c are fitting parameters. This type of evaluation can provide information on rupture times for a given set of applied stress and temperature. For example, equation (2) suggests that the rupture times under 137 MPa are approximately 4700 h at 1300 °C and 400 h at 1400 °C. There are cases where engineering design necessitates knowing the length of time required to reach a certain maximum level of strain, e.g. 1%. Therefore, in Fig. 3(b), the creep data is presented as *σ* vs. the Larson-Miller parameter, ${P}_{\mathrm{LM} \mbox{-} 1 \% }=T\cdot ({\rm{a}}+\,\mathrm{log}\,{t}_{1 \% })$, ${P}_{\mathrm{LM} \mbox{-} 1 \% }$ represents the temperature-compensated time required to reach the 1% strain. This plot succeeds in bringing the 1% creep data measured at 1400, 1500, and 1600 °C close to a single line, which can be expressed as:

3 $$\mathrm{log}\,\sigma ={\rm{b}}\cdot {P}_{\mathrm{LM} \mbox{-} 1 \% }+{\rm{c}}$$

Equation (3) also predicts that the 1% strain under 137 MPa will be reached in approximately 66 h at 1300 °C and approximately 5 h at 1400 °C. The fitting parameters (a, b, and c) for the two Larson-Miller plots shown in Fig. 3(a,b) are given in Table 2. Figure 3(c) shows that a general trend can be identified despite considerable scatter, and indicates that rupture strains increase with decreasing stress (dashed line). In Fig. 3(d), the minimum creep rates, $\dot{\varepsilon }$_*min*_, determined as shown in Fig. 2(b), are related to the rupture times by plotting the original Monkman-Grant relation^29^ (minimum creep rate vs. rupture time, open symbols, MG) and the modified relationship proposed by Dobeš and Milička^30^ (minimum creep rate vs. rupture time/rupture strain, filled symbols, DM). As seen in Fig. 3(d), the original Monkman-Grant plot (open symbols) has considerable scatter. In contrast, the inclusion of the rupture strains significantly improves the correlation of all data (filled symbols), which can be described by the linear equation shown in the figure.

**Table 2:** Dimensionless fit parameters a, b, and c for the master curves in the Larson-Miller plots shown in Fig. 5a (rupture) and 5b (strain limit: 1%).

Data type	a	b	c
Rupture	14.4	−0.155	6.542
1%	16.9	−0.201	8.056

**Figure 4:**
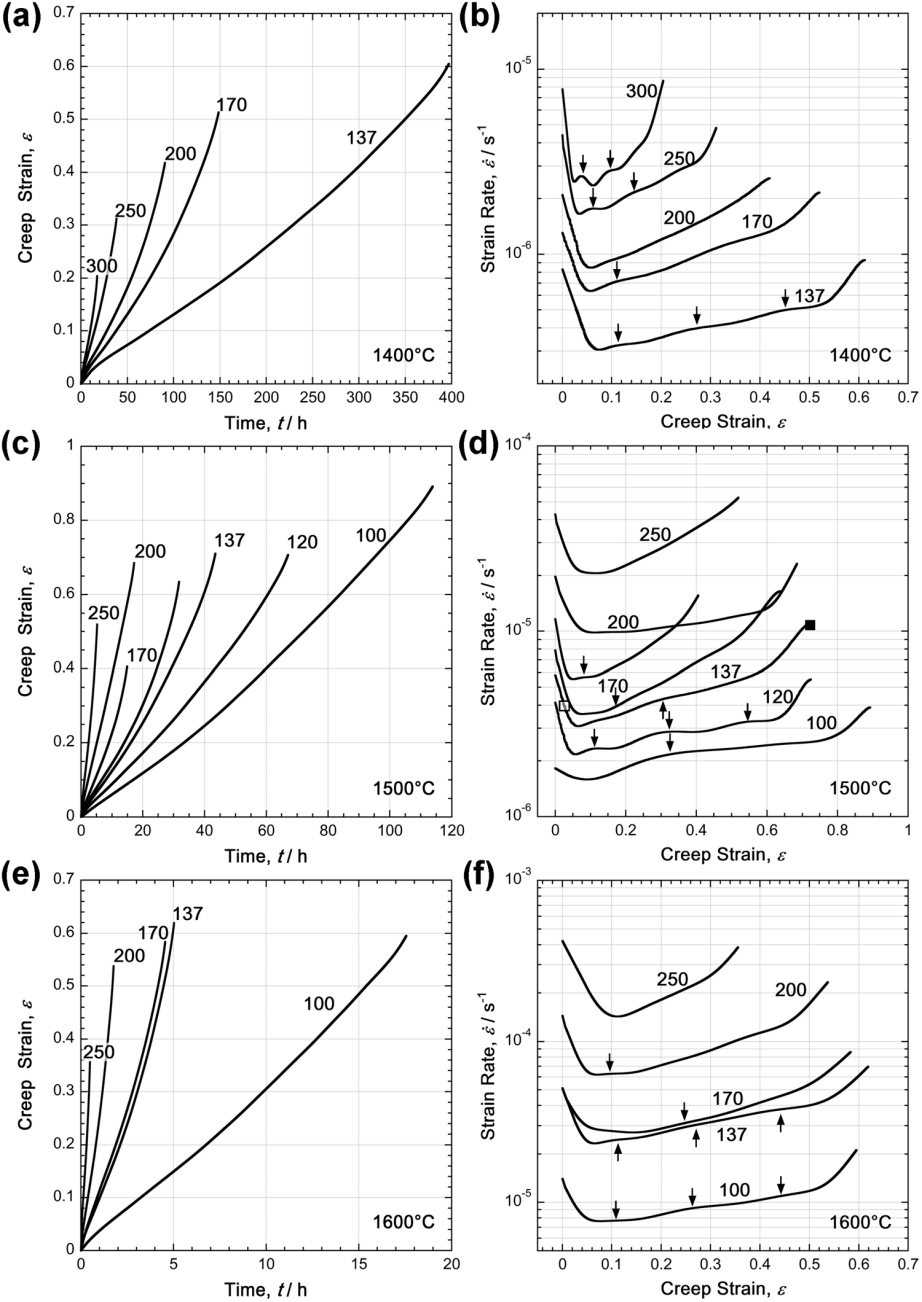
Experimental creep data presented as strain vs. time (h) (*ε*(*t*) curves) in (a,c,e) and as logarithmic strain rate vs. strain ($\mathrm{log}\,\dot{\varepsilon }(\varepsilon )$ curves) in (b,d,f). (**a**) and (**b**): 1400 °C, (**c**) and (**d**): 1500 °C, (**e**) and (**f**): 1600 °C. The numbers next to the curves indicate tensile stresses in MPa. Only one experimental condition was applied two times (170 MPa at 1500 °C). The curves are shown in (c,d). One experiment (137 MPa, 1500 °C, *ε* = 2.9%) was interrupted in the very early stages of creep (end of curve marked with an open square) and subsequently investigated with SEM. The end of an experiment which proceeded through rupture under the same creep conditions (137 MPa, 1500 °C, *ε*_R_ = 72%) is marked with a filled square. This specimen was also investigated with SEM.

Figure 4 shows the creep data for the experiments performed in this study. Figure 4(a,c,e) show the *ε*(*t*) creep curves and Fig. 4(b,d,f) show $\mathrm{log}\,\dot{\varepsilon }(\varepsilon )$ curves. For each temperature, the *ε*(*t*) curves are presented in the left column of Fig. 4 (a: 1400 °C; c: 1500 °C; e: 1600 °C), while the $\mathrm{log}\,\dot{\varepsilon }(\varepsilon )$ curves are presented in the right column (b: 1400 °C; d: 1500 °C; f: 1600 °C). $\mathrm{log}\,\dot{\varepsilon }(\varepsilon )$ curves all show primary creep regions where the creep rate decreases. At 1400 °C, the creep rate minima occur at strain levels between 2% and 6%. With increasing temperature, the minimum creep rates occur at increasing strains, i.e. 4–10% for 1500 °C and 5–13% for 1600 °C. In all cases, the strain rates increase only moderately after the minimum creep rates are reached until the very end of creep, at which point a significant increase in creep rate toward the final rupture is observed. Although it is easy to identify the end of the primary creep, separating the secondary and tertiary creep regimes is difficult.

Two features of the creep curves in Fig. 4 should be highlighted. First, for all test temperatures, the highest rupture strains accumulated at the lowest stresses, as explained in Fig. 3(c). Second, some of the creep curves exhibit small wavy features characterized by small local minima and maxima, which are marked by black arrows. These phenomena will be discussed later.

**Figure 5:**
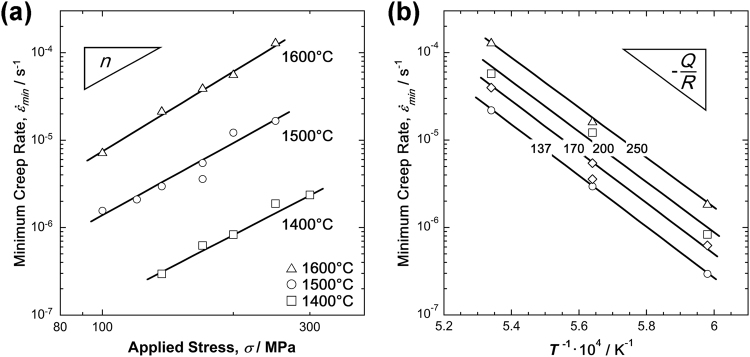
Stress and temperature dependence of the minimum creep-rate data based on equation 1. (**a**) Norton plot: minimum creep rate (s^−1^) as a function of stress (MPa) in a log-log plot. (**b**) Arrhenius plot: minimum creep rate (s^−1^) as a function of the inverse temperature (K^−1^).

From the creep data shown in Fig. 4, minimum creep-rate data could be determined, as shown in Fig. 2. The stress and temperature dependence of the minimum creep rates are described by equation (1), and the results are shown in Fig. 5(a,b). Figure 5(a) shows a Norton plot, in which the logarithm of the minimum creep rates from all the tests are plotted as a function of the logarithm of the applied stress. The minimum creep-rate data can be effectively rationalized with a Norton-type creep law having a stress exponent, *n*, close to 3. In Fig. 5(b), the minimum creep-rate data are presented in an Arrhenius plot, in which the natural logarithm of the minimum creep rate is plotted as a function of the inverse absolute temperature (in K). At all stress levels, the data can be effectively rationalized by the apparent activation energy of creep of 550 kJ/mol indicated in Fig. 5(b).

**Figure 6:**
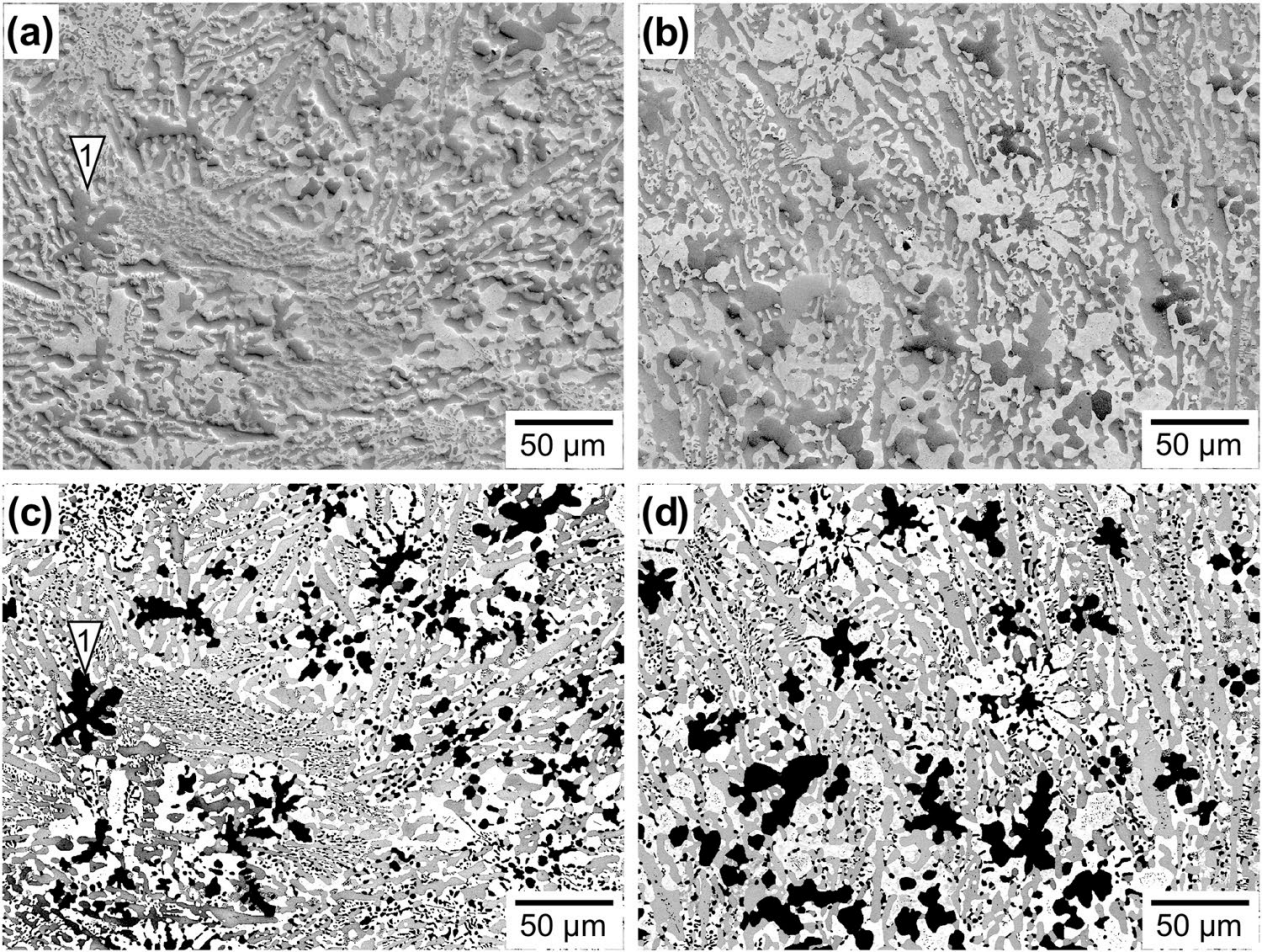
Effect of temperature exposure on the microstructure. SEM images were taken from the undeformed specimen grip sections for the experiments marked with open and filled squares in Fig. 4(d). (**a**) and (**c**) 1500 °C, 4.3 h, taken at the same location. (**b**) and (**d**) 1500 °C, 44 h, taken at the same location. (**a**) and (**b**) Secondary electron (SE) images. (**c**) and (**d**) Backscattered electron (BSE) images.

In order to interpret the mechanical data on a microstructural basis, three microstructural results are presented. Figure 6 shows SEM images of microstructures in the undeformed grip section of two specimens from creep experiments performed at 1500 °C and 137 MPa: one specimen is from a test that was interrupted early, and the other was allowed to continue until rupture. The microstructure shown in Fig. 6(a,c) was exposed to 1500 °C for a short period of 4.3 h. The features of the black (Ti,Mo)C particles did not change significantly compared to the initial state shown in Fig. 1(a). A large (Ti,Mo)C particle on the left of the micrograph is marked with an arrow; it shows the same dendritic features as the particle marked with an arrow and labelled “1” in Fig. 1(a) after processing and heat treatment. After 44 h of exposure (Fig. 6(b,d)), the microstructure had evolved; the well-defined dendritic features were no longer apparent, and the (Ti,Mo)C particles coalesced and appeared coarser. Moreover, the T_2_ phase regions also appeared to have coarsened. In contrast, the Mo_ss_ regions showed little change and appeared to be as fine as before. Figure 6(a,b) show that no creep cavities were detected in the undeformed parts (grip sections) of the creep specimens deformed at 1500 °C and 137 MPa.

**Figure 7:**
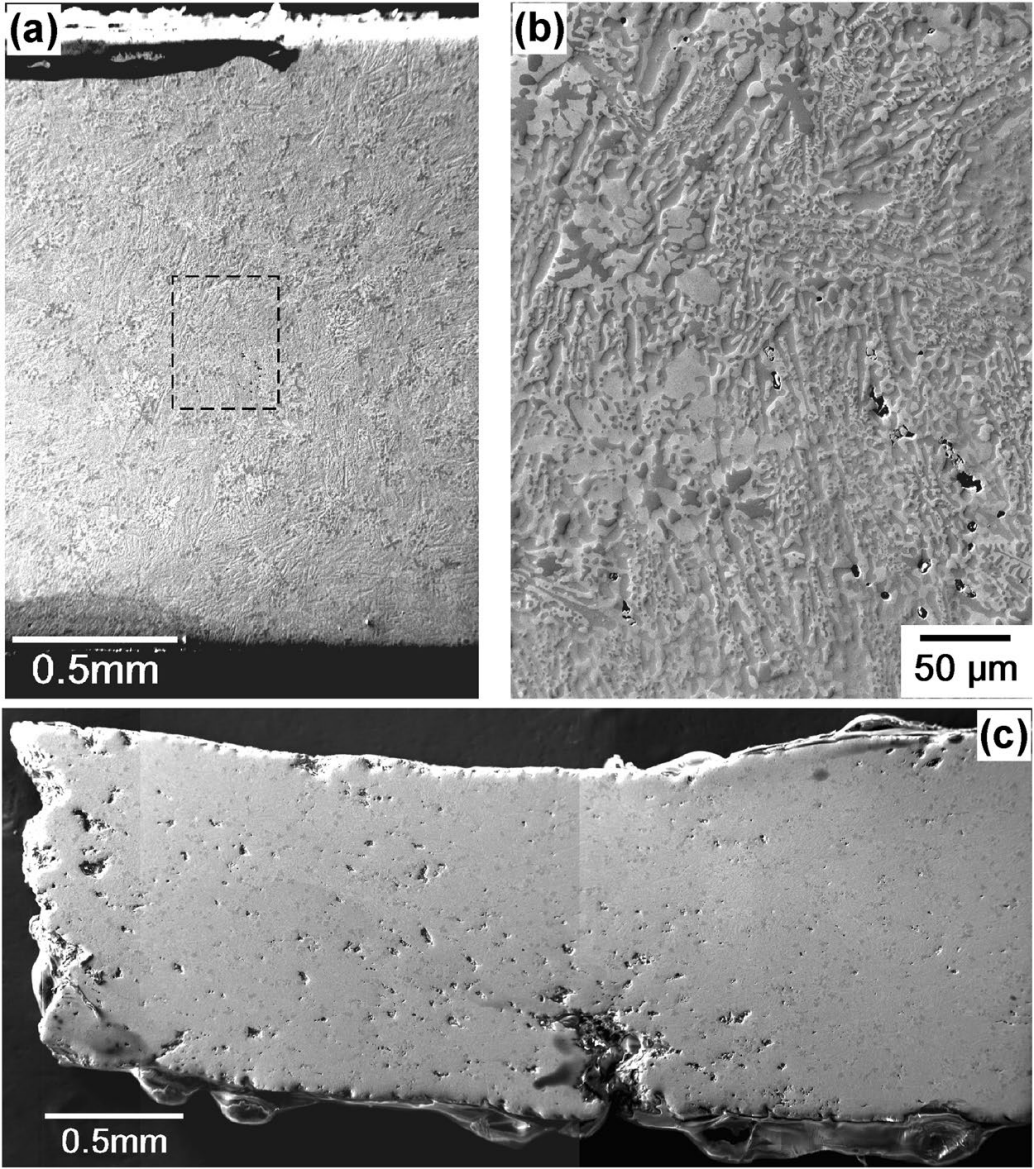
SEM image montages taken after creep deformation at 1500 °C and 137 MPa. (**a**) Overview montage of SEM micrographs after short creep exposure (2.9%, 4.3 h). (**b**) Higher magnification SEM image from the region indicated by the dashed rectangle in Fig. 7(a) showing that small pores form in the early stages of creep. (**c**) Overview montage of SEM micrographs after rupture (72%, 44 h).

Figure 7 shows SEM images from the gauge lengths of the two specimens after creep at 1500 °C and 137 MPa. The specimens were polished and investigated using secondary electron SEM contrast. Figure 7(a) shows an SEM image of the gauge length after short creep exposure. Here, the cavity sizes are small and the overall cavity density is low. Small cavities which have formed in the location of the microstructure indicated by the dashed rectangle in Fig. 7(a) are shown at a higher magnification in Fig. 7(b); the nucleation and growth of creep cavities are shown to start very early in creep life. After rupture, a high density of large cavities is found in the microstructure, as illustrated in Fig. 7(c). The presence of large cavities close to the rupture surface on the left of the image suggests that the creep rupture initiated in regions where a group of cavities interlinked and formed a microcrack. The large crack in the lower part of the specimen likely represents a location where the creep damage reached a similar level as in the region where the final rupture occurred.

**Figure 8:**
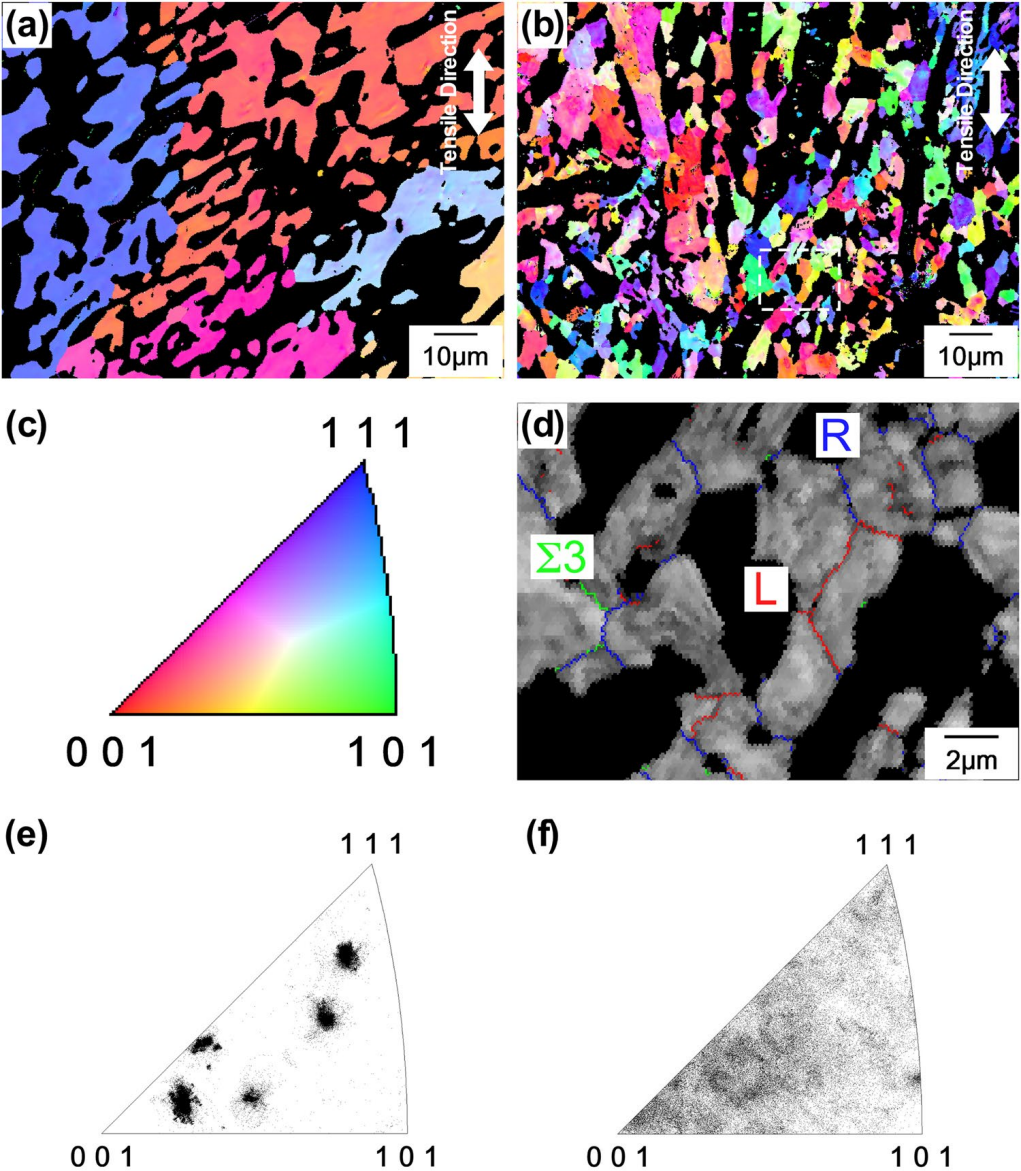
Colour-coded IPF maps of Mo_ss_ phase in the alloy obtained when looking on the specimen surfaces after short and longer creep exposure at 1500 °C and 137 MPa. The orientation distributions of grains are shown in a view direction parallel to the tensile axis. (**a**) Short creep exposure (2.9%, 4.3 h), showing a large connected Mo_ss_ region with similar orientations. (**b**) Longer creep exposure (72%, 44 h) obtained at the same magnification as Fig. 8(a). (**c**) The [001]-[101]-[111] standard triangle for cubic structures showing colour-coded crystallographic orientations. (**d**) Longer creep exposure, the same as Fig. 8(b), showing large connected Mo_ss_ regions having very small grains with high and low angle grain boundaries. (**e**) and (**f**) Inverse pole figures of Mo_ss_ phase corresponding to Fig. 8(a,b), respectively.

The two material states shown in Fig. 7(a,b) (short-term creep exposure, creep curves shown in Fig. 4(c,d)) and Fig. 7(c) (longer-term creep exposure, creep curves shown in Fig. 4(c,d)) are also depicted in Fig. 8. Figure 8(a,b) show the colour-coded inverse pole figure (IPF) maps of the Mo_ss_ phase in the alloy taken in the view direction of the specimen surface normals using electron backscatter diffraction pattern (EBSD)^31^. The orientation distributions of Mo_ss_ grains are in the view direction of the tensile axis, and those of other phases are shown as black regions. The crystallographic standard triangle of Fig. 8(c) shows the colour coding used for specific crystallographic directions^31^. Figure 8(a) reveals that in the early stages of creep, percolating connected regions of the Mo_ss_ phase with similar orientations exist and extend beyond 10 µm. The region in Fig. 8(a) shows four orientation types, which are shown in tangerine (near <001>), blue (close to <111>), pink (close to <113>), and light blue (orientation in the interior of the standard triangle). From the EBSD observations of several regions including Fig. 8(a), it can be concluded that the Mo_ss_ phase shows no texture. During creep, the microstructural features of the Mo_ss_ phase change significantly. The connected regions of the Mo_ss_ phase still exist in the microstructure, which do not change in size and shape. However, instead of large connected regions with similar orientations, a fine grain structure with grain sizes of a few micrometers is observed, as shown in Fig. 8(b). Figure 8(d) shows that these small grains have low- and high-angle grain boundaries. In addition, the inverse pole figure maps in Fig. 8(a,b) demonstrate that the orientation distribution of Mo_ss_ grains was almost random even after the long-term. This result strongly suggests that dynamic recrystallization is predominant in the Mo_ss_ phase in the present creep conditions. The EBSD analyses also examine the volume fractions of the constituent phases in the interrupted creep specimens. It should be noted here that the results show scatter in the range of ±3% with no systematic trend as regards strain, suggesting that the volume fractions of the constituent phases hardly changed during long-term creep exposure.

## Discussion

An objective of this study was to provide a first set of creep data for the MoSiBTiC alloy for temperatures of 1400–1600 °C and stresses between 100 and 300 MPa. The method used in this study involves a creep test under vacuum. In this ultrahigh-temperature range, a computer-controlled electromechanical test rig was used to monitor creep tests with rupture lives of up to 400 h. Constant true-stress creep tests were performed by feeding the digital displacement signal back into the control circuit, and reproducible creep data were obtained showing a scatter not larger than that observed during low-temperature creep tests, as shown in Figs 2–4. The rupture plots in Fig. 3 and the minimum creep-rate plots in Fig. 5 show that the creep results are self-consistent, i.e. higher strain rates and shorter rupture times are obtained at higher stresses and higher temperatures. Throughout the temperature and stress ranges under consideration, the minimum creep rates show a stress and temperature dependence that can be characterized by a stress exponent, *n*, of 3 and a *Q*_*app*_ of 550 kJ/mol. It is interesting to compare these creep activation parameters with results reported in the literature. For this purpose, the results from high-temperature mechanical experiments and diffusion experiments are compiled in Table 3.

**Table 3:** Compilation of activation parameters *n* and *Q*_*app*_ (high-temperature plasticity) and *Q*_*d*_ (diffusion data) reported in literature. The following abbreviations are used: TC, tensile creep; CSRT, constant strain-rate tensile testing; CC, compression creep; TD, tracer diffusion experiments.

Year of Publ.	Literature Source	Alloy Composition in at.%	Experiment Type	Temp. Range (°C)	*n*	*Q*_*app*_ in kJ/mol
1979	Handbook^55^	Mo	TC	2000–2500	—	214
2001	Nieh *et al*.^56^	Mo-9.4Si-13.8B	CSRT	1400–1450	2.8	740
2007	Ciulik and Tallef ^57^	Mo	CSRT	1340–1540	5	234–246
2007	Jéhanno *et al*.^58^	Mo-8.9Si-7.7B (HIP)	CC	1050–1315	2.0	444
		Mo-8.9Si-7.7B (annealed)			2.3	477
2010	Jain and Kumar^25^	Mo-3.0Si-1.3B	TC	1000–1300	5.5	360
2014	Hochmuth *et al*.^33^	Mo-9Si-8B	TC	1100–1250	3	560
		Mo-9Si-8B-2Zr Mo-9Si-8B-4Zr				460
2014	Schliephake *et al*.^34^	Mo-12.5Si-8.5B-27.5Ti	CC	1200–1300	3–4	560
		Mo-9Si-8B-29Ti				384
2014	Hasemann *et al*.^59^	Mo-17.5Si-8B	CC	1093–1400	3.4–4.4	391
2016	Hasemann *et al*.^60^	Mo-17.5B-10B	CC	1093–1400	3–4.5	318
1979	Maier *et al*.^32^	Mo	TD	1087–2500	-	460

Table 3 lists results for pure Mo and a number of different Mo-based alloys subjected to various mechanical tests, including tensile creep testing (TC), compression creep testing (CC), and constant strain-rate tensile testing (CSRT). This table also includes a value for the self-diffusion of ^99^Mo in Mo^32^. Maier *et al*.^32^ reviewed the diffusion results reported by other researchers, which agreed reasonably well with their data. Here, it should be highlighted that the apparent activation energy for creep determined in this study is not very different from the Mo diffusion value reported by Maier *et al*.^32^. On the other hand, our activation energy is also on the same order of magnitude as the activation energies reported by Hochmuth *et al*.^33^ for Mo-9Si-8B and Mo-9Si-8B with the addition of 2% and 4% Zr, and by Schliephake *et al*.^34^ for Mo-12.5Si-8.5B-27.5Ti and Mo-9Si-8B-29Ti. The results reported by Schliephake *et al*.^34^ suggest that small differences in chemical composition can have a significant effect on creep activation parameters. Unfortunately, as far as we have surveyed, reasonable activation energy values for the impurity diffusion of Si and Ti in Mo have not yet been reported. For the activation energy of creep in Mo-Si-B-based alloys, more careful discussion of impurity diffusion and interstitial (I)-substitutional (S) impurity interaction effects may be needed^35,36^. Most of the authors listed in Table 3 reported stress exponents between 3 and 5. Today, it is well known that the stress exponent, *n*, does not necessarily have a well-defined physical meaning^37^. However, in the early days of creep research, a stress exponent of 3 was considered to describe a steady-state creep based on the viscous motion of dislocations^38^. Straub and Blum have shown that there are problems with this basic interpretation, even for pure Al^39^. The microstructural results reported in Figs 6–8 indicate that the minimum creep rates observed in this study cannot be rationalized as steady-state creep, in which a dynamic equilibrium between work hardening and time softening establishes a constant dislocation substructure. Our material consists of several phases that evolve during creep. Jain and Kumar^25^ investigated a less complicated Mo-3.0Si-1.3B-type alloy at temperatures of 1000–1300 °C. Their creep activation parameters (*n* = 5.5, *Q*_*app*_ = 360 kJ/mol) differ from our findings, probably as a result of differences in the alloy composition and the temperature and stress ranges under consideration. To rationalize their two-phase system, they used simple models^40,41^ developed for coarse microstructures and based on a rule of mixtures. This approach does not seem to be appropriate for rationalizing the results obtained in the present study, because our microstructure is very fine and the individual phases interact in a specific manner which will be discussed later.

An important mechanical finding of the present study is that creep rupture strains tend to increase with decreasing stress, as shown in Fig. 3(c). In materials that exhibit creep cavitation, an opposite trend is observed: high stresses favour fast plastic deformation, and cavity nucleation, growth, and interlinkage do not occur, while at low stresses, a cavitation-based damage mechanism can operate, resulting in smaller rupture strains. This, is observed in tempered martensite ferritic steels, for example^42^. Our material showed nucleation and growth of cavities, which then formed microcracks and initiated the final rupture, as shown in Fig. 7. Nevertheless, rupture strains increased with decreasing stress, reaching values of up to 70% (Figs 3(c) and 4).

From a mechanistic point of view, this suggests a mechanism that exhibits some of the characteristics of the superplastic material behaviour of fine-grained and nanocrystalline materials^43–46^. In fine-grained materials, grain boundary sliding contributes significantly to the overall strain accumulation. Several elementary processes are involved in a manner that can be complex even for single-phase materials. It seems reasonable to assume that grain boundary diffusion plays a role in the ultrahigh-temperature creep of the MoSiBTiC alloy; however, a stress exponent value of 3 indicates that the creep mechanism is not pure Coble creep, as reviewed in the article^47^. Grain boundary sliding requires accommodation processes^43–46^. For the MoSiBTiC alloy, phase boundary sliding should be more precisely discussed because T_2_/Mo_ss_ and (Ti,Mo)C/Mo_ss_ phase boundaries are involved. The T_2_ and (Ti,Mo)C phases appear to act as stiff components of the microstructure even though they undergo Ostwald ripening-type coarsening^48,49^, as shown in Figs 6 and 7. In our multicomponent system, this requires that the diffusion of T_2_ and (Ti,Mo)C-forming elements can occur in the Mo_ss_ phase. More importantly, the Mo_ss_ phase undergoes dynamic recovery and dynamic recrystallization. This can be concluded from the moderate strain-rate oscillations indicated by the small arrows in Fig. 4. These correspond to stress oscillations, which have been reported for constant strain rate tests in the classical theories of dynamic recrystallization^50,51^. The EBSD data shown in Fig. 8 also represent microstructural evidence for dynamic recovery and recrystallization events in the Mo_ss_ phase.

Larson-Miller parameter plots are important to formulate and predict the creep rupture life, *t*_R_, and they are also often applied to predict times which it takes to reach specific strains. The Larson-Miller constant denoted as ‘a’ in the present study has been often found to be close to 20^52^, and this value was also used for the tensile creep of Mo-Si-B alloys by Jain and Kumar^25^. On the other hand, a Larson-Miller constant of 13.42 which is much lower than 20 was successfully used to rationalize creep data from commercial heat-resistant molybdenum alloys such as TZM and MHC^53^. The values of 14.4 for the rupture life and 16.9 for the 1% accumulated strain limit estimated in the present study (Fig. 3) may also suggest that the creep strain of the MoSiBTiC alloy is governed by the creep deformation behaviour of the Mo_ss_ phase in the alloy. The Larson-Miller relationship and the Monkman-Grant and Dobeš and Milička relationships should be continuously studied for the MoSiBTiC alloy to obtain better creep life predictions by taking grain boundary and interfacial sliding, void formation, dynamic recovery and recrystallization of Mo_ss_ into account.

In summary, mechanical and microstructural results from the ultrahigh-temperature creep testing of a TiC-reinforced Mo-Si-B-based alloy are reported in this paper. This study indicates that the MoSiBTiC alloy has great potential as an ultrahigh-temperature material. The results can be summarized as follows:

1. Creep tests on the MoSiBTiC alloy were performed at temperatures between 1400 and 1600 °C and stresses between 100 and 300 MPa for up to 400 h using a computer-controlled test rig with an integrated vacuum chamber. Flat dog-bone specimens were tested using a graphite grip system integrated in the load line. The load cell signal of the test rig and the displacement value were used by the control unit to enforce constant true stress test conditions. The scattering in the resulting creep data is lower than that for creep tests performed at lower temperatures.
2. The creep rupture data set obtained in this study can be phenomenologically rationalized using Larson-Miller and Monkman-Grant-type plots. At high temperatures, creep nucleation and growth of cavities were observed, which led to the formation of microcracks and eventually initiated final rupture. Nevertheless, rupture strains increase with decreasing stresses.
3. A large portion of the creep life is spent at the minimum creep rate. Throughout the stress and temperature ranges under consideration, the stress and temperature dependence of the minimum creep rate could be described by a stress exponent, *n*, of 3 and a *Q*_*app*_ of 550 kJ/mol. These activation parameters are discussed based on previous work with related materials including pure Mo. The stress exponent of 3 allows us to exclude the possibility that creep is solely diffusion-controlled.
4. Fine strain-rate oscillations in the creep curves and microstructural refinement in the Mo_ss_ phase of the MoSiBTiC alloy microstructure detected by orientation imaging using a scanning electron microscope suggest that dynamic recovery and dynamic recrystallization are the key elementary processes during ultrahigh-temperature creep.
5. The creep mechanism governing the ultrahigh-temperature creep of the MoSiBTiC alloy involves a combination of coupled microstructural processes. Cavities nucleate and grow continuously; however, this process is not very intense and does not result in a pronounced tertiary creep behaviour. Although the T_2_ and (Ti,Mo)C phases undergo coarsening, they appear to act as hard regions which are connected by the Mo_ss_ phase. The high rupture strains indicate grain boundary sliding between the Mo_ss_ phase and the two types of hard regions, and reveal that the Mo_ss_ phase accommodates high plastic deformation by undergoing dynamic recovery and recrystallization. Further work is required to clarify this aspect.

## Methods

In the present study, we investigated a MoSiB alloy with Ti and C additions, which has a nominal chemical composition of 65Mo-5Si-10B-10Ti-10C (at%). The material was prepared following an ingot metallurgy processing route from feedstock consisting of pure Mo (99.99%), Si (99.9999%), B (99.95%), and cold-pressed TiC powder (99% purity, grain size: 2–5 µm). First, 90 g of 45 mm diameter button ingots were prepared by arc melting (five re-melting cycles) under a protective argon atmosphere using a water-cooled Cu crucible. The as-cast material was then subjected to a homogenization heat treatment at 1800 °C for 24 h in an argon atmosphere. The microstructure evolution of this material during processing and creep has been described elsewhere^12,18^. The microstructure was observed using a JEOL JSM-7800F scanning electron microscope operating in the backscatter electron (BSE) mode. Scanning electron microscopy (SEM) was also used to study the effect of creep on microstructure. Secondary electron SEM was used to clarify whether creep cavities formed, and orientation imaging SEM (EBSD)^31^ was used to clarify whether any changes on the grain level of the Mo_ss_ phase can be detected after creep.

**Figure 9:**
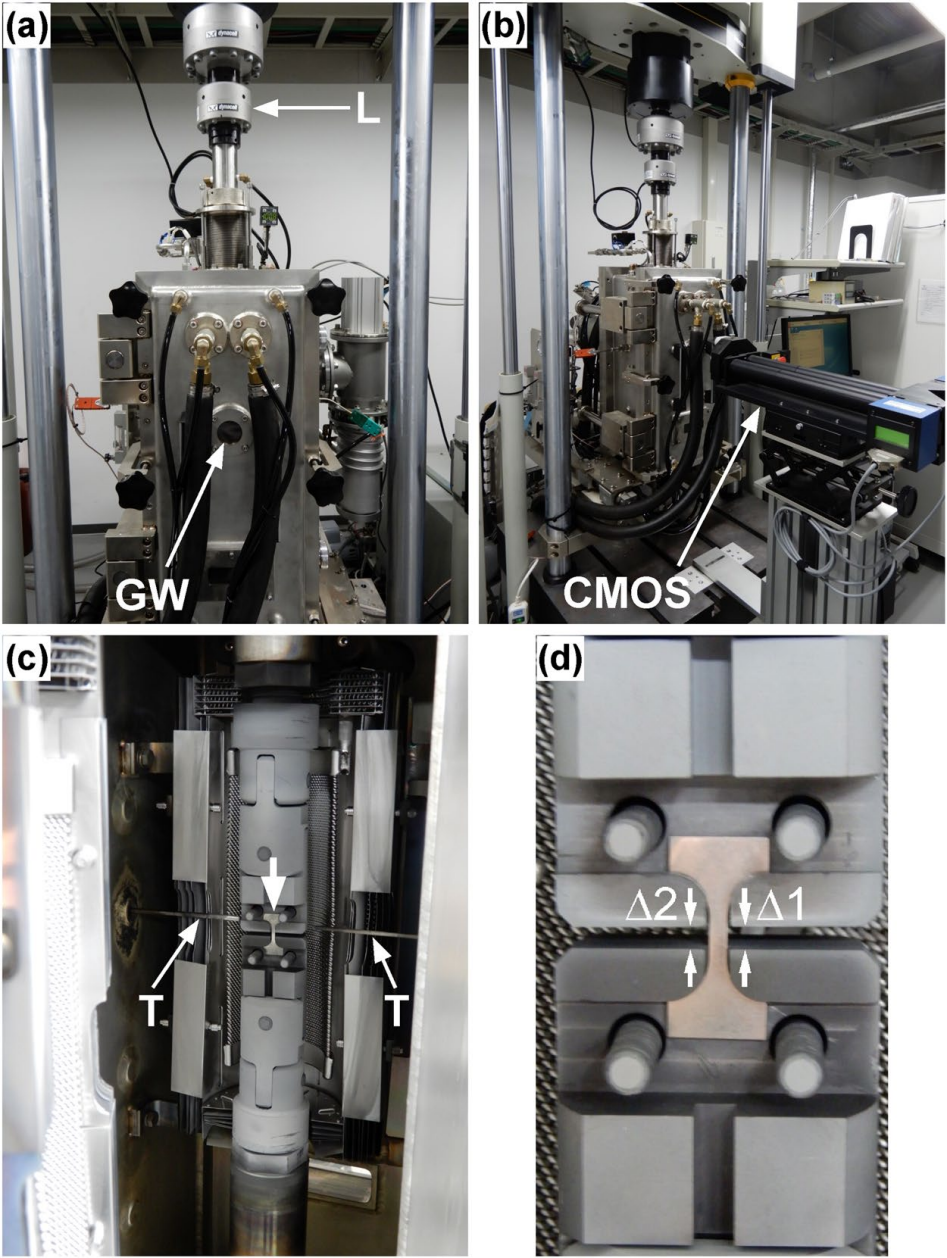
High-temperature test rigs used in this study. (**a**) Closed loop test system with a vacuum chamber; the load cell (L) and front glass window (GW) are highlighted. (**b**) Extensometer system placed in front of the GW. (**c**) Open vacuum chamber with a specimen (down arrow) and thermocouples (T) highlighted. (**d**) A specimen and graphite loading grips. The widths of the two gaps, Δ1 and Δ2, are recorded during creep testing.

**Figure 10:**
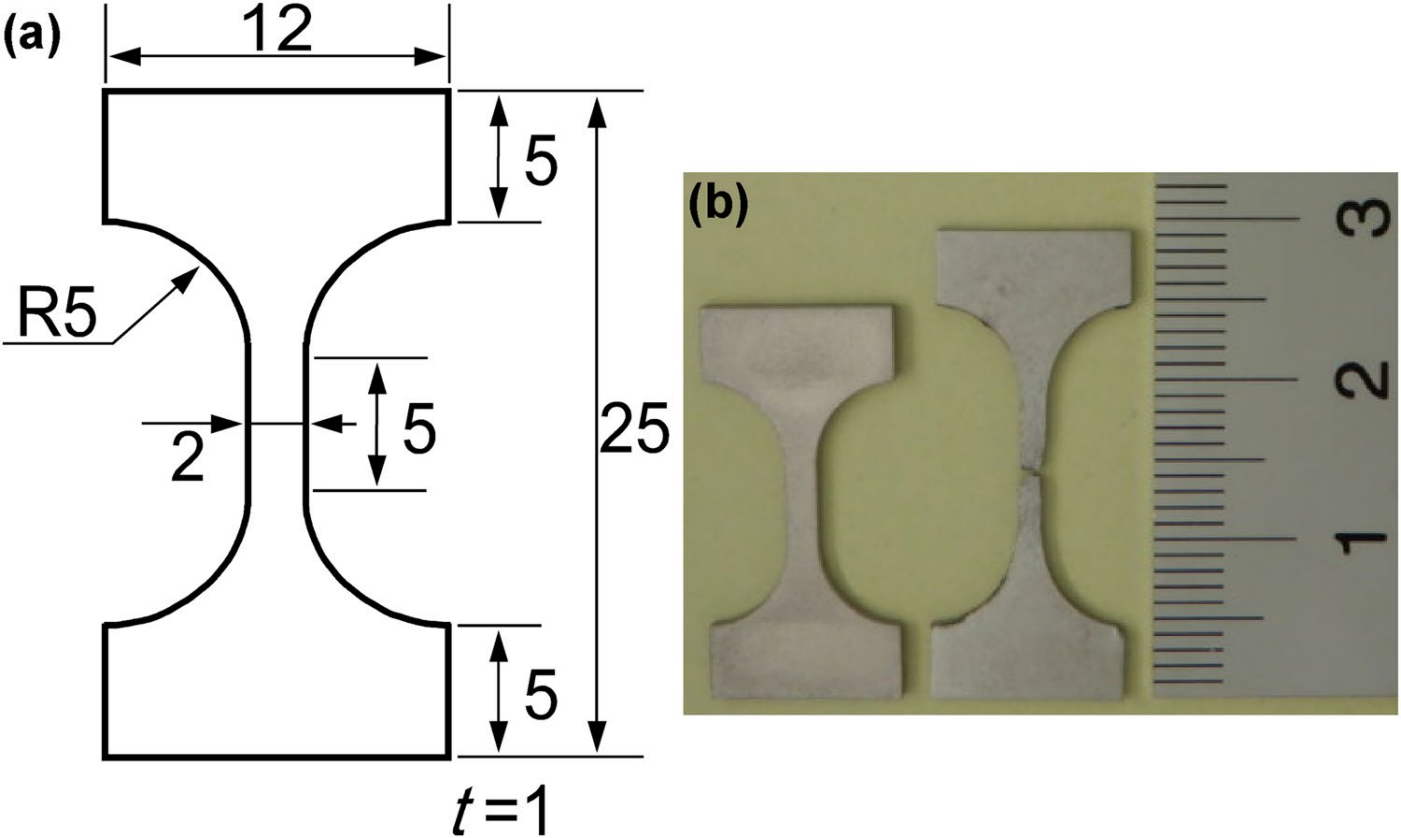
Creep specimen used in this study. (**a**) Geometry of a flat dog-bone specimen (all dimensions given in mm). (**b**) Creep specimens before and after creep testing. Creep rupture occurred at the centre of the gauge length.

For ultrahigh-temperature requirements, a special uniaxial creep test setup and procedure were developed, as shown in Fig. 9. Figure 9(a) shows a closed-loop Instron 8862 type control test rig with an integrated vacuum furnace (Thermonic, Tokyo, Japan), which enabled load-controlled tensile creep tests at a vacuum of greater than 10^–3^ Pa. The load cell (marked ‘L’) can be seen above the vacuum chamber. There are two glass windows on both sides of the chamber; only the front window can be seen and is indicated by an arrow. Figure 9(b) shows two metal-oxide ZS16AHS type semiconductor line sensors (indicated by CMOS in Fig. 9(b)), which send signals through the two glass windows on the emitter (GW, Fig. 9(a)) and receiver (not shown) sides. A digital camera on the other side of the vacuum chamber (not shown) is connected to a system that can evaluate the data and transform the distance measurements into electric signals (system from H.D. Rudolph GmbH, Germany). These signals can be fed back into the control system through the dynamic testing control and acquisition software, WaveMatrix, provided by Instron. In the open vacuum chamber shown in Fig. 9(c), one side of the heating system consisting of two W-mesh resistance heater elements can be seen. The tensile specimen in the centre of the image is indicated by a downward arrow. Figure 9(c) also shows the upper and lower elements of the load line, which induce a mechanical load on the specimen. In Fig. 9(c), the upper part of the graphite grip system is connected to the upper loading bar, and two horizontal thermocouples (indicated by arrows and labelled ‘T’) can be seen. The graphite grip system is an original design for the ultrahigh-vacuum tensile creep testing, and is machined from high-quality graphite by TOYO TANSO, Osaka, Japan. Figure 9(d) shows the grip system with an integrated specimen outside the furnace for illustration purposes, and indicates the widths of two gaps, Δ1 and Δ2, between the upper and lower grips. The widths of these gaps can be measured with the digital extensometer system shown in Fig. 9(b). The average value, Δ = (Δ1 + Δ2)/2, is used for the strain calculation, by measuring the elongation of the specimen, Δ(*t*), at time *t* as the difference between Δ1(*t*) and Δ1(0). The recording and control system evaluates the strain, *ε*_0_, by dividing Δ(*t*) by the gauge length, *l*_0_, of the dog-bone specimen, which is shown in Fig. 10. Figure 10(a) shows the specimen geometry (all dimensions in mm). Figure 10(b) shows a specimen before and after the tensile creep test. Note that when an ultrahigh-temperature creep test is continued until failure, the tensile specimen fails in the middle of the gauge length, as required for a valid creep test. After spark erosion machining from the heat-treated ingots, the specimens were ground and polished to a mesh size of 1500. During testing, displacement data were continuously measured, and creep strains were obtained ${\varepsilon }_{0}={\rm{\Delta }}/{l}_{0}$. Assuming a constant volume during plastic deformation, the true stress $\sigma$, can be expressed as a function of the applied stress ${\sigma }_{0}=P/(2\,{{\rm{mm}}}^{2})$, and the engineering strain ${\varepsilon }_{0}={\rm{\Delta }}(t)/(5\,\mathrm{mm})$, by the well-known $\sigma ={\sigma }_{0}\cdot (1+{\varepsilon }_{0})$^54^. Implementing this relation into the WaveMatrix application software allowed us to maintain a constant true stress during the experiment.
